# Talking during walking: the diagnostic potential of turn dynamics in Alzheimer’s disease, mild cognitive impairment and cognitive aging

**DOI:** 10.3389/fnagi.2025.1533573

**Published:** 2025-02-19

**Authors:** Hedieh Mohammadi, Adel Maghsoudpour, Maryam Noroozian, Fatemeh Mohammadian

**Affiliations:** ^1^Department of Mechanical Engineering, Science and Research Branch, Islamic Azad University, Tehran, Iran; ^2^Cognitive Neurology, Dementia, and Neuropsychiatry Research Center, Tehran University of Medical Sciences, Tehran, Iran; ^3^Department of Psychiatry, Roozbeh Hospital, Tehran University of Medical Sciences, Tehran, Iran

**Keywords:** gait analysis, dual-task test, turn dynamics, cognitive aging, Alzheimer’s disease, mild cognitive impairment, dementia

## Abstract

**Background:**

While gait analysis is well-documented, turn performance—which is a more complex task and involves multiple brain regions—has been less explored. This study aims to assess the diagnostic potential of turn dynamics as a novel tool for detecting cognitive decline.

**Methods:**

We recruited 75 participants, including 26 neurotypical (NT) older adults, 25 with amnestic mild cognitive impairment (aMCI), and 24 with mild Alzheimer’s disease (AD). Participants completed a dual-task walk and turn (DTWT) test using a dual Kinect setup while counting backwards by ones. Key measures analyzed included spatial-temporal parameters of gait and turn dynamics. Statistical analyses including analyses of variance and linear regression were performed to identify key features as well as to assess their correlation with cognitive performance.

**Results:**

Gait speed and stride time significantly differentiated among groups in DTWT conditions. More notably, turn dynamics, particularly segmental peak speeds and step length, displayed stronger discriminatory power with more significant *p*-values compared to gait features. Linear regression analysis indicated that turn dynamics had stronger correlations with executive function and working memory, suggesting a more pronounced relationship between cognitive performance and turn features than gait variables.

**Conclusion:**

In contrast to straight walk metrics, this study shows that DTWT turn dynamics are more sensitive to detect cognitive impairment. Consequently, incorporating turning movements into gait analysis techniques could enhance diagnostic protocols in clinical settings, offering a valuable tool for monitoring the progression of conditions associated with cognitive aging.

## 1 Introduction

Alzheimer’s disease (AD), accounting for an estimated 60 to 80% of all dementia cases (Kapasi et al., 2017), imposes significant social and healthcare costs around the world. Research indicates that early-stage detection can potentially slow down the advancement of dementia or mitigate its effects (Montero-Odasso et al., 2017). Consequently, identifying mild cognitive impairment (MCI), as a transitional phase between normal age-related cognitive decline and dementia is crucial (Chen et al., 2022). Therefore, a non-invasive and effective clinical marker capable of detecting cognitive decline in the early stages is essential.

Motor function requires the coordination of the brain’s cortical, subcortical, and cerebellar regions; any deficits in these areas can impair motor performance (Yogev-Seligmann et al., 2008). Research has demonstrated that motor deficits, which are prevalent up to 12 years before a dementia diagnosis, can serve as a valuable diagnostic tool for early cognitive decline (Montero-Odasso et al., 2009a; Beauchet et al., 2013). Dual-task gait tests have shown promising results which, follows from increased activity in higher-level cortical regions, namely the pre-frontal cortex (PFC) (Ramirez and Gutierrez, 2021; Ali et al., 2024). The competitive demands for cognitive resources lead to the unveiling of hidden motor deficits in patients with dementia (Montero-Odasso et al., 2018). Moreover, small vessel disease affects motor function neuroanatomical substrates, causing dementia-related movement impairments. White matter loss in this condition can disrupt brain connections, affecting cognitive and motor abilities (Sharma et al., 2023). However, most gait analysis research for cognitive impairment has focused on straightforward walking patterns with cognitive activities potentially overlooking the additional demands of complex maneuvers. For example, research indicated that complex walking patterns may reveal subtle differences between patients with MCI and neurotypical individuals (Wang et al., 2024; Poosri et al., 2024; Seifallahi et al., 2024), one particularly underexamined maneuver is turning.

Turning is a complex task, as it necessitates the central nervous system to regulate body reorientation toward a new direction, while sustaining the ongoing step cycle and postural stability (Thigpen et al., 2000). While walking straight primarily relies on maintaining balance and rhythmic gait, turning requires precise spatial awareness, decision-making, planning, processing environmental cues and coordinating movement (Hase and Stein, 1999; Lowry et al., 2012; Lee and Park, 2018). Studies suggest that, the PFC is involved in deciding when and how to make a turn, planning its trajectory, timing and sequencing. The PFC also integrates visual, auditory, and proprioceptive information to guide turning behavior (Herman et al., 2011; King et al., 2012; Tavares and Tort, 2022). Additionally, the roles of the parietal cortex integrating sensory information for spatial awareness and the hippocampus supporting the spatial memory for navigation is crucial (Calton and Taube, 2009; Eichenbaum, 2017). The presence of advanced motor and cognitive processes increases the susceptibility of turning to cognitive deficits (Lee et al., 2022). Studies have shown that medial-lateral control, crucial for maintaining balance during turns, is decreased in older adults. Additionally, older individuals tend to take more steps and turn slower than younger adults (Baird and Van Emmerik, 2009; Conradsson et al., 2018; Khobkhun et al., 2021). Also, a reduced segmental angle and speed were observed in older adults during a turn as a compensatory mechanism (Khobkhun et al., 2021; Khobkhun et al., 2022a). A decrease in postural control may be ascribed to impairments in motor system functions; or the capacity to carry out cognitive activities (Baird and Van Emmerik, 2009). For instance, research suggests a correlation between attention and executive function in older adults and their ability to maintain balance during a turn (Tangen et al., 2014). Moreover, timed up and go (TUG) test, similar to walking and turning, is showed to be linked to working memory and visuospatial ability (Mirelman et al., 2014; Ansai et al., 2018).

Despite the recognized importance of turning movements, research gaps persist in objectively quantifying turn dynamics in patients with cognitive impairment. Addressing this, our study analyzes walk and turn aspects of AD, MCI and, neurotypical individuals to identify significant features that distinguish three groups. Next, we analyze the correlation between key variables and participants’ cognitive performance.

## 2 Materials and methods

### 2.1 Materials

This section details the materials and resources utilized to conduct the study.

#### 2.1.1 Participants and clinical assessment

**FIGURE 1:**
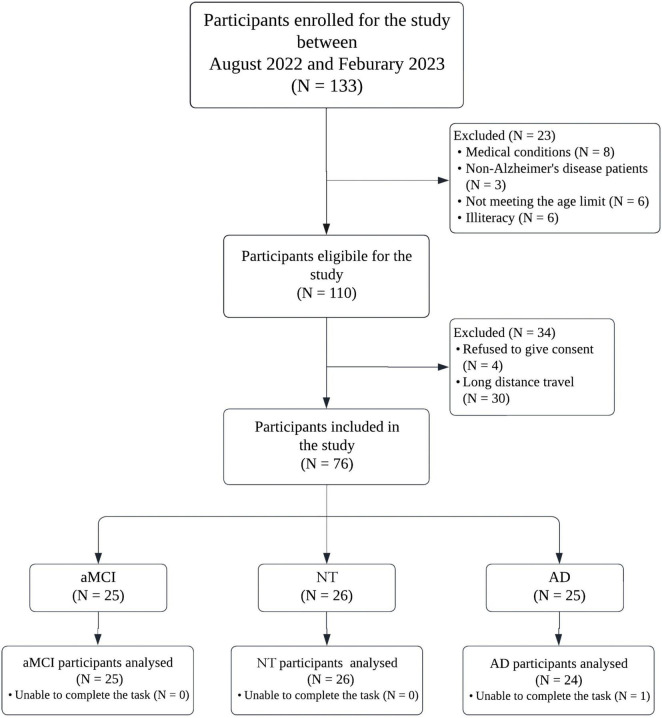
Study flow chart. AD, Alzheimer’s disease; NT, neurotypical; aMCI, amnestic mild cognitive impairment.

In this study, 26 neurotypical (NT) older adults, 25 patients with amnestic-MCI (aMCI), and 24 patients with mild AD, over 65 years old participated. Patients with AD and aMCI were recruited from national referral centers: Yaadmaan; institute for Brain, Cognition and Memory Studies and the cognitive neurology and neuropsychiatry division and the department of psychiatry, Roozbeh hospital, Tehran University of Medical Sciences. Healthy older adults were notified via advertisements in local communities. This study was approved by the Research Ethics Committees of Islamic Azad University Science and Research Branch (Approval ID: IR.IAU.SRB.REC.1401.300); Informed consent was obtained directly from each participant prior to the test, including aMCI and NT individuals. For patients with AD, the consent was provided by legal guardians. It is worth noting that the test was non-invasive and safe for all participants. Figure 1 shows the flow diagram of the study.

For all participants two neurologists expert in dementia and cognitive disorders conducted a consistent clinical assessment protocol. Patients diagnosed with AD were assessed using the clinical diagnosis of AD criteria established by the Alzheimer’s disease and related disorders association and the national institute of neurological and communicative disorders and stroke (ADRDA/NINCDS) (McKhann et al., 1984). NT older adults and patients with aMCI were assessed with neuropsychological examination and cognitive assessment tests. Data regarding participants’ demographic information, clinical results related to co-morbidities, and familial dementia histories were gathered. Cognitive functions for all groups, were evaluated using the mini-mental state examination (MMSE) and Montreal cognitive assessment (MoCA) tests (Folstein et al., 1975; Nasreddine et al., 2005). MoCA is a screening tool designed to assess various cognitive domains and is particularly effective in identifying MCI and early stages of AD while, MMSE is commonly employed to assess individuals with moderate to severe cognitive impairments (Pinto et al., 2019). A higher cognitive ability is indicated by a total score of 30 points for both tests. If an individual has less than 12 years of education, one extra point will be added to their initial MoCA score (Shirk et al., 2011). For assessing attention and working memory the serial 7s test was utilized in which the participants were asked to do a series of subtractions 7 from 100 (Manning, 1982; Bristow et al., 2016). For evaluating the executive function, planning and visuospatial abilities the clock drawing test (CDT) was administered in which individuals were asked to draw the face of a clock, put in all the numbers, and set the hands to 10 after 11 using paper and a pencil (Sunderland et al., 1989). For both tests the MoCA scoring (0–3) were utilized (Nasreddine et al., 2005). Additionally, for the subset of participants diagnosed with aMCI and AD the functional assessment staging tool (FAST) was conducted (Sclan and Reisberg, 1992; Noroozian et al., 2022). FAST is an assessment method developed to evaluate the impairment of functional abilities in patients with AD at all stages of the condition. FAST scale assigns a score that measures the gradual decline of functional abilities across seven key levels. For our research, we used clinical data obtained from participants’ regular medical checkups, which took place within one week before their involvement in the study.

The study excluded individuals with neurological disorders including cerebral stroke; Parkinson’s disease; multiple sclerosis; cerebral palsy; peripheral neuropathy; musculoskeletal problems such as hip or knee prosthesis; lower limb or hip fractures; severe osteoporosis or arthritis; muscle weakness; serious cardiopulmonary problems; or other medical conditions that could affect gait. Additionally, individuals with a history of falls in the past six month; or ongoing drug or alcohol abuse; or those taking any medications known to alter gait, were not considered. The study specifically excluded patients with moderate to severe stages of AD (FAST > 5) and other types of dementia, as these patients fell outside the study’s scope. In addition, participants had to have the ability to walk a distance of 10 meters and make a 180-degree turn unassisted by any mobility aids. Individuals who need glasses or hearing aids were expected to utilize them.

#### 2.1.2 Sample size

The sample size was calculated using G*Power 3.1 software, based on statistical power at 80% and one-way α level of 0.05 using the analysis of variance (ANOVA) test with the effect size of 0.4 based on the reference study’s gait speed feature of TUG test (de Oliveira Silva et al., 2020). The choice was due to the similarities in the populations being studied and the aspects of mobility being assessed. At least 66 participants were required to acquire the total sample. The estimated power of this study was above 0.818. To enhance the robustness and compensate for potential missing data, we recruited beyond this minimum.

#### 2.1.3 Experiment’s setup

Motion data were recorded with two Microsoft Kinect v2 depth sensors (Microsoft Corporation, Redmond, WA, USA, 2015). The Kinect v2 can track up to 25 joints per person with a sample rate of 30 frames per second. The operational range is from 0.5-meters to 4.5-meters, with a field of view of 70 degrees horizontally and 60 degrees vertically and the depth resolution is 512 × 424 pixels.

**FIGURE 2:**
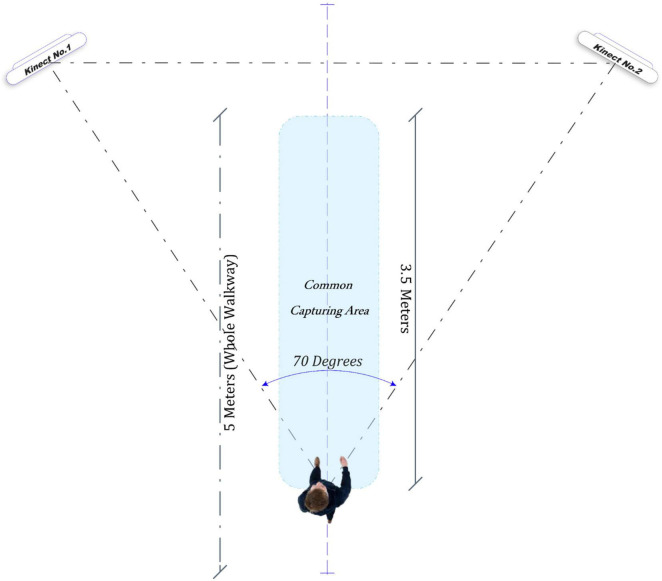
Experiment’s setup.

In establishing the configuration for the dual Kinect setup, an initial assessment evaluated angles ranging from 60 to 180 degrees. The objective was to maximize the capturing area while ensuring adequate coverage of turning movements. Our evaluation indicated that angles within the 60 to 90 degrees range were optimal for our needs. Subsequently, within this range, a series of trial-and-error led to cameras positioned at 70 degrees relative to each other and 1.18-meters above ground height, both directed toward the designated capture area: a walkway with 5 m length and 0.6-meters width. Within this span, a 3.5-meters segment fell directly within both cameras field of view. Figure 2 illustrates the specifics of this setup.

Calibration, recording, and motion tracking procedures were executed using iPi Motion Capture software version 4.5.8.260 (iPi Soft, LLC, Moscow, Russia). To guarantee synchronized data capture from the cameras they were connected to a shared wireless network. Detailed information for these procedures is available in Supplementary materials (see Supp1, eFigure1 and eFigure2).

#### 2.1.4 Walk-and-turn test

Dual-task walk and turn (DTWT) test: After receiving the “start” command, cameras began recording and participants walked the 5 m walkway, turned around, and returned at a self-selected pace while counting backwards by ones starting from 100. The chosen arithmetic task is commonly used in dual-task walk investigations and its effects have been confirmed in previous studies (Montero-Odasso et al., 2009b; Cullen et al., 2018). We chose counting by ones rather than a more difficult subtraction (e.g., by sevens) to accommodate the broad range of educational levels in our sample, ensuring the task remained feasible and consistent for all participants.

Although the entire walkway is used for the DTWT test, analysis focused on the 3.5-meter section captured by the cameras. This helped with steady-state data capturing, as the initial and final 1.5 meters are prone to gait disturbances due to starting and stopping movements.

Prior to the assessment, participants were advised to avoid wearing black clothing due to the Kinect sensor’s sensitivity to black colors. To ensure accurate body movement capture, tight clothing and closed-toe comfortable shoes were also required. Additionally, the walkway was covered with mats to reduce floor reflections, enhancing ground detection accuracy. Furthermore, the test area was evaluated for participant safety, ensuring sufficient lighting and a flat surface free of obstacles. All tests were conducted in the same room and setup at Yaadmaan center.

### 2.2 Data analysis

#### 2.2.1 Feature extraction

The task given to participants was segmented into two distinct phases: the “walk” phase and the “turn” phase. Commonly, healthy young and older adults employ a craniocaudal sequence of movements to change direction while walking. This starts with the rotation of head, followed by the trunk, and then the pelvis in the yaw plane (Fuller et al., 2007; Khobkhun et al., 2021). However, considering that patients with cognitive impairment may have altered movement sequences, the initiation of the turn phase was carefully marked as the point when the first body segment began its turn and feet, head, and hip were taken as body segments. The turn phase concluded once the last body segment completed its turning motion. All other movements were categorized under the walk phase.

For each body segment, the onset of the turn sequence was identified when its yaw angle began to exceed the average yaw angle fluctuation observed during the forward walk. In the context of this study, average fluctuations refer to the typical, minor variations in the yaw angle of a body segment (such as the head or hip) observed during straight walking. The completion of the turn for that body part was marked when its yaw angle realigned with the average fluctuation seen during the return walk. Each phase’s start and end points were also thoroughly investigated frame by frame. The iPi Studio software tracked human body movements and generated the skeleton data. The Cartesian coordinates and Euler angles for the following joints were extracted: head, hip, left foot, right foot, and the center of mass (CoM). A total of 15 dependent variables were calculated from the skeleton data.

Parameters of walk phase: stride length; stride time; gait speed; double support time (DST); cadence; stride time variability (STV); swing phase and stance phase were calculated using the methodologies established in previous studies (Cullen et al., 2018; see Supp1, eTable1 in Supplementary materials). The mean values of the walk phase parameters were reported for the forward and return trips, to mitigate potential variability which may be introduced by environmental factors.

**FIGURE 3:**
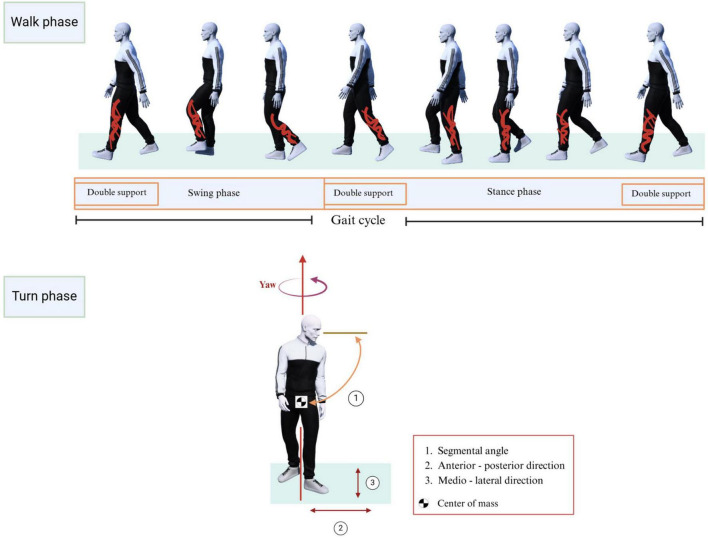
Walk phase (one gait cycle illustrated) and turn phase parameters.

A total of seven parameters were derived for turn phase: turning time is defined as the duration between the onset and the end of the turning sequence; step length was calculated as the average anterior-posterior distance between consecutive heel strikes during the turn phase; Peak head and hip speeds were calculated as the maximum angular speeds of head and hip segments in the yaw direction (Khobkhun et al., 2021); peak segmental angle represents the maximum angular difference between the head and hip segments during a turn (Khobkhun et al., 2021); sway-AP and sway-ML were calculated as the difference between maximum and minimum values of the anterior-posterior (AP) and the medio-lateral (ML) directions of the CoM signal. Figure 3 walk and turn phases’ parameters.

#### 2.2.2 Statistical analysis

To describe the population, Chi-square test–for nominal variables–and the Kruskal–Wallis and one-way ANOVA tests were utilized for group comparisons, followed by the Tukey HSD and Mann–Whitney U tests for pairwise comparisons.

The distribution of gait features was evaluated using the Shapiro–Wilk test; the Levene’s test was used to check the homogeneity of variance. Several features were identified to be non-normally distributed thus, they underwent Box-Cox power transformation method. For primary analysis, one-way ANOVA test with a Bonferroni correction was undertaken. To identify feature differences among groups while mitigating the influence of confounders, we performed an analysis of covariance (ANCOVA) to control for covariates. Based on the previous research, in this study the potential covariates were considered as sex, age, years of education, and body mass index (BMI) (Bruce-Keller et al., 2012; Gomes Gde et al., 2015; Montero-Odasso et al., 2017; Jayakody et al., 2018; Pieruccini-Faria et al., 2021; Bovonsunthonchai et al., 2022). Subsequently, a Tukey honestly significant difference (HSD) test was employed as a *post hoc* analysis for features that appeared significant. To achieve accurate and stable calculations the confidence intervals (CI) of η^2^ (eta squared) as a measure for effect size were employed to ensure the precision and stability of estimations. For calculating the CI of η^2^, we employed stratified bootstrapping to maintain the natural distribution of data using 1,000 iterations to estimate the 95% CI for eta squared. This method involved resampling individual group data with replacement, followed by recalculating eta squared for each iteration. The CIs were then derived from the 2.5th and 97.5th percentiles of the bootstrapped eta squared distributions. The η^2^ values were reported based on Cohen’s effect size guideline (Cohen, 2019).

To investigate whether cognitive functions were associated with gait and turn features under the DTWT test, we conducted univariate linear regression analyses using the ordinary least squares (OLS) method. Each significant gait or turn feature served as the dependent variable in the regression. We then created two models to capture different aspects of cognitive function. Model 1 utilized CDT scores as predictor, treated as an ordinal variable. CDT provides an assessment of planning, visuospatial ability, and aspects of executive function, which are relevant for gait and turn performance (Yogev-Seligmann et al., 2008). Model 1 considers age and years of education as covariates. Model 2 examines the association between gait and turn features and serial 7s scores with the same covariates as Model 1. The Serial 7s test heavily involves working memory and attention processes that may influence DTWT test (Salzman et al., 2025). The key distinction between Model 1 and Model 2 is the primary cognitive measure used as the predictor. Both models control for the same covariates (age and education) to account for potential demographic influences on gait and cognition. For each model the regression coefficients, *p*-values, and Shapley additive explanations (SHAP) values, illustrating the relative importance of each predictor in influencing gait and turn parameters, were reported. SHAP value quantifies how much each predictor contributes, either positively or negatively, to the target variable compared to the prediction’s baseline. All analyses were conducted using Python version 3.11.5, the SciPy version 1.11.1, (Virtanen et al., 2020) and Scikit-Learn version 1.3.0, (Pedregosa et al., 2011).

## 3 Results

### 3.1 Participants’ characteristics

Table 1 summarizes the demographics for each group. There were no significant differences in sex distribution with 53% female or BMI (*p*-value = 0.56) across the three groups. However, age and years of education showed significant disparities. The NT group was younger on average (69.3 ± 4.4) compared to the aMCI (73.8 ± 5.5) and AD (74 ± 5.3) with *p*-value = 0.04. Additionally, participants in the NT group had more years of education (15.3 ± 4.9) than those with AD (10.5 ± 5.6) with *p*-value < 0.001. As a result, years of education and age were considered as covariates in this study. As expected, MoCA and MMSE scores differed significantly among the three groups (*p* < 0.001), reflecting their distinct levels of cognitive function. Although smoking showed a significant difference between groups (*p* = 0.04), it was not included in the ANCOVA test because, we opted to limit the model complexity by including only those covariates that are most directly linked to cognitive function and gait performance.

**TABLE 1:** Participants’ characteristics.

Variable	Cognitive statues			Test results	
	NT (*n* = 26) Mean ± SD	aMCI (*n* = 25) Mean ± SD	AD (*n* = 24) Mean ± SD	(*p*-value)	*Post hoc*
Female, *n* %^b^	11 (42.3)	14 (56)	15 (62.5)	0.43	–
Age in years^a^	69.3 ± 4.4	73.8 ± 5.5	74 ± 5.3	0.04*	NT > AD
Years of education^a^	15.3 ± 4.9	12.4 ± 3.5	10.5 ± 5.6	< 0.001*	NT > AD
MMSE (0–30)^a^	28.5 ± 1.1	25.5 ± 2.6	18.6 ± 4.5	< 0.001*	NT > AD aMCI > AD aMCI < NT
MoCA (0–30)^a^	25.6 ± 1.3	21.9 ± 2.6	13.6 ± 4.2	< 0.001*	NT > AD aMCI > AD aMCI < NT
CDT (0–3)^c^	2.6 ± 0.6	2.0 ± 0.8	1.7 ± 1.0	< 0.001*	NT > AD
Serial 7s test (0–3)^c^	2.6 ± 0.4	2.2 ± 0.9	1.2 ± 1.0	< 0.001*	NT > AD aMCI > AD
Height (m)^a^	1.6 ± 0.0	1.5 ± 0.7	1.5 ± 0.1	0.29	–
Body weight (Kg)^a^	75.1 ± 11.3	69.1 ± 11.5	69.5 ± 13.3	0.12	–
BMI ($\frac{K\,g}{m^{2}}$)^a^	29 ± 4.1	27.7 ± 4.1	28.1 ± 4.1	0.56	–
Hypertension, *n* %^b^	14 (53)	14 (56)	13 (54)	0.98	–
Hyperlipemia, *n* %^b^	6 (23)	6 (24)	8 (33)	0.66	–
Diabetes, *n* %^b^	5 (21)	9 (36)	5 (20)	0.32	–
Smoking, *n* %^b^	6 (23)	5 (20)	0 (0)	0.04*	NT > AD
CAD, *n* %^b^	5 (19)	6 (24)	5 (20)	0.91	–
Depression, *n* %^b^	6 (23)	7 (28)	4 (16)	0.63	–

AD, Alzheimer’s disease; aMCI, amnestic mild cognitive impairment; BMI, body mass index; CAD, coronary arterial disease; CDT, clock drawing test; MMSE, mini-mental state examination; MoCA, Montreal cognitive assessment; NT, neurotypical; SD, standard deviation.

*^a^*One-way ANOVA test, *Post hoc*: Tukey HSD.

*^b^*Chi squared test, *Post hoc*: Pairwise comparisons.

*^c^*Kruskal–Wallis test, *Post hoc*: Mann–Whitney U.

*p-value < 0.05.

### 3.2 DTWT parameters comparison analysis

The results of DTWT test, shown in Table 2, also revealed disparities in gait and turn dynamics in the ANOVA and ANCOVA tests with years of education and age and covariates. The CI lower bound of eta squared is reported as η^2^; the choice of reporting the lower bound of CI was due to the variability observed in the CIs so the lower bound of these intervals was reported as a conservative estimate of effect size. This cautious strategy ensures that our conclusions are robust against potential sampling variability and helps prevent the overestimation of effect sizes. Significant reduction in stride length was detected as cognitive impairment increased; and the difference remained significant after conducting the ANCOVA test (*p*-value = 0.02), however, the *p*-value increased notably. Stride time also varied significantly among groups (η^2^ = 0.11, *p*-value < 0.001) with AD group having longer stride time compared to other groups. STV was observed to be higher in the group with AD compared to other groups. In addition, this difference was accompanied by a moderate effect size (η^2^ = 0.1, *p*-value < 0.001), showing an increase in gait irregularity but this feature appears to be more affected by covariates. The differences in gait speed were notable, as indicated by the effect size and *p*-value (η^2^ = 0.29, *p*-value < 0.001). Also, gait speed was the only parameter in the walk phase able to distinguish all groups. Results showed AD group had less cadence than others. DST increases correlated with cognitive impairment, however, after correcting for variables these differences were not significant. No variations were seen in swing and stance phases.

**TABLE 2:** DTWT parameters comparison analysis.

Variable	Cognitive status			Test results		
	NT (*n* = 26) Mean ± SD	aMCI (*n* = 25) Mean ± SD	AD (*n* = 24) Mean ± SD	η^2^ (adj. *p*-value)^b^ ANOVA	(adj. *p*-value)^c^ ANCOVA	*Post hoc*
**Walk phase**						
Stride length (m)^a^	1.1 (0.6)	1.0 (0.9)	0.9 (0.8)	0.13 (*p* < 0.001)*	(*P* = 0.02)*	NT > AD aMCI < NT
Stride time (s)	1.2 ± 0.1	1.3 ± 0.1	1.4 ± 0.1	0.11 (*p* < 0.001)*	(*p* = 0.003)*	NT < AD aMCI < AD
STV (%)^a^	6.4 (9.7)	7.0 (14.7)	12.4 (24.9)	0.1 (*p* < 0.001)*	(*p* = 0.02)*	aMCI > AD NT < aMCI
Gait speed (m/s)	1.0 ± 0.1	0.7 ± 0.1	0.6 ± 0.1	0.29 (*p* < 0.001)*	(*p* < 0.001)*	NT > AD aMCI > AD aMCI < NT
Cadence (Step/min)	95.2 ± 8.2	93.6 ± 11.6	82. ± 9.1	0.1 (*p* < 0.001)*	(*p* = 0.009)*	NT > AD aMCI > AD
DST (s)	0.1 ± 0.04	0.2 ± 0.04	0.2 ± 0.05	0.04 (*p* = 0.02)*	(*P* = 0.5)	–
Swing phase (%)^a^	0.3 (0.3)	0.3 (0.1)	0.3 (0.1)	(*p* = 0.06)	–	–
Stance phase (%)^a^	0.6 (0.1)	0.6 (0.1)	0.6 (0.06)	(*p* = 0.6)	–	–
**Turn phase**						
Turning time (s)^a^	1.9 (1.5)	2.0 (2.1)	2.9 (2.8)	0.13 (*p* < 0.001)*	(*p* = 0.006)*	NT < AD aMCI < AD
Step length (m)^a^	0.3 (0.3)	0.1 (0.4)	0.1 (0.3)	0.21 (*p* < 0.001)*	(*p* < 0.001)*	NT > AD aMCI > AD
Peak head speed (°/s)	453.6 ± 104.6	345.1 ± 154.2	237.3 ± 65.3	0.26 (*p* < 0.001)*	(*p* < 0.001)*	NT > AD aMCI > AD MCI < NT
Peak hip speed (°/s)^a^	441.9 (367.7)	320.9 (640.6)	238.6 (213)	0.31 (*p* < 0.001)*	(*p* < 0.001)*	NT > AD aMCI > AD MCI < NT
Peak segmental angle (°)	41.4 ± 11.3	30.3 ± 11.8	24.7 ± 11.5	0.13 (*p* < 0.001)*	(*p* < 0.001)*	NT > AD aMCI < NT
Sway-AP (m)	0.5 ± 0.2	0.4 ± 0.1	0.3 ± 0.1	0.11 (*p* < 0.001)*	(*p* = 0.006)*	NT > AD aMCI > AD
Sway-ML (m)^a^	0.1 (0.2)	0.1 (0.3)	0.1 (0.2)	(*p* = 0.8)	–	–

AD, Alzheimer’s disease; aMCI, amnestic mild cognitive impairment; ANOVA, analysis of variance; ANCOVA, analysis of covariance; AP, anterior-posterior; DST, double support time; ML, medio-lateral; NT, neurotypical; SD, standard deviation; STV, stride time variability.

*^a^*Median (Min-Max range)—Normalized.

*^b^*One-way ANOVA with Bonferroni correction, *Post hoc*: Tukey HSD.

*^c^*ANCOVA with Bonferroni correction—years of education and age were included as covariates.

*p-value < 0.05.

Considering the turn phase, turning time was significantly different, with NT participants taking less time to finish a turn compared to aMCI and AD after the ANCOVA test (*p*-value = 0.006). Noticeable reductions in the length of steps during a turn were seen (η^2^ = 0.21, *p*-value < 0.001). aMCI and AD groups had significantly lower peak head and hip speeds than NT people, with considerable effect sizes. After adjusting for confounders, both variables remained significant, suggesting they may discriminate groups. Even after controlling for confounders, individuals with aMCI and AD had a lower peak segmental angle during turns than NT participants (*p*-value < 0.001). Sway-AP also differed significantly across groups, with decreased sway observed as cognitive impairment increased. However, no differences were observed in the sway-ML measurements among the groups. It is worth noting that turn phase features were able to stay significant even after considering the effect of covariates in the analysis.

### 3.3 Association between cognitive performance and walk and turn features

Table 3 indicates the results for two univariate linear regression models after adjusting for years of education and age. Model 1 investigates the correlation between walk and turn parameters and the CDT scores in order to assess the relation between DTWT features and spatial awareness and planning. While model 2 evaluates the association between these same characteristics and serial 7’s scores which evaluates different cognitive domains such as working memory and attention through repeated subtraction tasks. Regarding walking Phase only stride length demonstrated a significantly positive relationship for both models (Coeff_model1 = 0.23 and Coeff_model2 = 0.32). Features like stride time, gait speed, and cadence have demonstrated higher coefficients and level of significance in Model 2 suggesting that participants with stronger working memory could more effectively manage the motor demands of walking while performing a cognitive task. STV did not demonstrate significant relation with cognitive scores.

**TABLE 3:** Linear regression analysis results.

Variable	Coeff.	*R*^2^ *adj*.	*P*-value	F-statistics	Coeff. (95% CI)
**Walk phase**					
Stride length (m)	**Model 1:** 0.23 **Model 2:** 0.32	**Model 1:** 0.32 **Model 2:** 0.25	**Model 1:** 0.03* Model 2: 0.005*	**Model 1:** 12.5 **Model 2:** 12.8	**Model 1:** [0.02, 0.45] **Model 2:** [0.09, 0.54]
Stride time (s)	**Model 1:** −0.14 **Model 2:** −0.38	**Model 1:** 0.04 **Model 2:** 0.14	**Model 1:** 0.25 **Model 2:** 0.002*	**Model 1:** 2.06 **Model 2:** 7.45	**Model 1:** [−0.41, 0.1] **Model 2:** [−0.62, −0.14]
STV (%)	**Model 1:** 0.04 **Model 2:** −0.15	**Model 1:** 0.02 **Model 2:** 0.03	**Model 1:** 0.72 **Model 2:** 0.23	**Model 1:** 1.52 **Model 2:** 1.98	**Model 1:** [−0.21, 0.3] **Model 2:** [−0.4, 0.1]
Gait speed (m/s)	**Model 1:** 0.18 **Model 2:** 0.39	**Model 1:** 0.21 **Model 2:** 0.24	**Model 1:** 0.11 **Model 2:** 0.001*	**Model 1:** 7.7 **Model 2:** 12.9	**Model 1:** [−0.04, 0.41] **Model 2:** [0.16, 0.62]
Cadence (Step/min)	**Model 1:** 0.13 **Model 2:** 0.38	**Model 1:** 0.03 **Model 2:** 0.13	**Model 1:** 0.32 **Model 2:** 0.002*	**Model 1:** 1.60 **Model 2:** 6.71	**Model 1:** [−0.12, 0.39] **Model 2:** [0.14, 0.62]
**Turn phase**					
Turning time (s)	**Model 1:** −0.2 **Model 2:** −0.37	**Model 1:** 0.14 **Model 2:** 0.19	**Model 1:** 0.09 **Model 2:** 0.009*	**Model 1:** 5.25 **Model 2:** 6.95	**Model 1:** [−0.4, 0.03] **Model 2:** [−0.55, −0.08]
Step length (m)	**Model 1:** 0.33 **Model 2:** 0.36	**Model 1:** 0.21 **Model 2:** 0.23	**Model 1:** 0.005* Model 2: 0.002*	**Model 1:** 7.84 **Model 2:** 8.51	**Model 1:** [0.10, 0.52] **Model 2:** [0.13, 0.59]
Peak head speed (°/s)	**Model 1:** 0.28 **Model 2:** 0.42	**Model 1:** 0.11 **Model 2:** 0.19	**Model 1:** 0.02* Model 2: 0.001*	**Model 1:** 4.20 **Model 2:** 7.04	**Model 1:** [0.03, 0.52] **Model 2:** [0.18, 0.66]
Peak hip speed (°/s)	**Model 1:** 0.23 **Model 2:** 0.43	**Model 1:** 0.14 **Model 2:** 0.24	**Model 1:** 0.056 **Model 2:** < 0.001*	**Model 1:** 5.03 **Model 2:** 8.94	**Model 1:** [−0.00, 0.42] **Model 2:** [0.21, 0.65]
Peak segmental angle (°)	**Model 1:** 0.32 **Model 2:** 0.29	**Model 1:** 0.11 **Model 2:** 0.1	**Model 1:** 0.01* Model 2: 0.022*	**Model 1:** 4.16 **Model 2:** 3.72	**Model 1:** [0.08, 0.55] **Model 2:** [0.05, 0.54]
Sway-AP (m)	**Model 1:** 0.08 **Model 2:** 0.31	**Model 1:** 0.04 **Model 2:** 0.11	**Model 1:** 0.49 **Model 2:** 0.014*	**Model 1:** 2.25 **Model 2:** 4.22	**Model 1:** [−0.11, 0.37] **Model 2:** [0.05, 0.55]

CDT, clock drawing test; Model 1, linear regression model with CDT score as predictor; Model 2, linear regression model with serial 7s score as predictor. Both models were adjusted for years of education and age.

*p ≤ 0.05.

Features in turn phase for both models suggest stronger significance and more association with cognitive tests. Turning time is the only feature which has a negative relation with serial 7s with a 0.37 coefficient value, indicating that better working memory facilitated quicker turns under dual-task conditions. For peak segmental angle and step length, the differences between models are not notable in comparison to other features. In addition, similar to walk phase, all features especially peak head and hip speed demonstrate more significant relation with serial 7s test according to the *p*-values and F statistics. Sway-AP shows significantly positive association in working memory.

**FIGURE 4:**
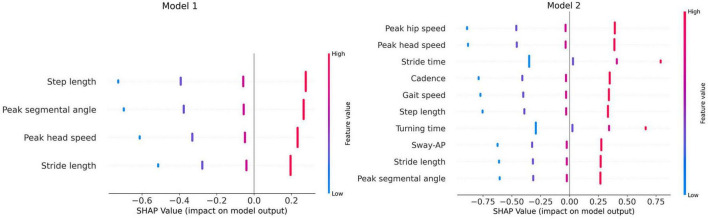
SHAP summary plots. AP, anterior-posterior, Model 1, linear regression model with CDT test as predictor; Model 2, linear regression model with serial 7s test as predictor.

Figure 4 illustrates the SHAP summary plots for features that had a significant relationship with predictor variables in both models, based on the univariate linear regression analysis results. SHAP plots excel at revealing how individual DTWT features influence model predictions, providing insights into both the overall importance and their specific impact on individual predictions. The horizontal axis represents the SHAP values and the vertical axis is the list of gait features ordered based on their impact on the model. Positive SHAP values suggests that the higher cognitive performance tends to push the predicted feature to higher values (red color code). In contrast, negative SHAP values indicate that higher cognitive performance leads to lower outcomes (blue color code).

According to model 1, step length and peak segmental angle have the most impact on the model and they also display positive SHAP values, suggesting that higher CDT scores are associated with longer steps and larger angles. Peak segmental angle shows that higher cognitive function (CDT scores) is associated with greater range of motion in body segments indicating that stronger visuospatial and planning abilities result in smoother turn. Peak head speed and stride length show red colored dots saturated on positive SHAP values, implying that higher CDT scores generally enhance these features values. This aligns with the notion that better cognitive function can be linked to faster walking and body segment speeds. In model 2, more features have a significant relationship with the predictor variable than in model 1, and larger SHAP values were observed for these features. In this plot, peak head and hip and stride time have the most correlation with serial 7s test; also, peak segmental angle has descended to the bottom of the list. Features like stride time and turning time have blue dots saturated on the left side of SHAP value axis which indicate that higher serial 7s scores result in lower values for these features. This confirms the assumption that serial 7’s scores as a metric for working memory tends to positively influence the speed of gait and turn parameters. On the contrary other features have higher feature values concentrated on the right side of the *x*-axis suggesting that patients with better serial 7s scores walk and turn faster with longer stride length and segmental angles. Additionally, the SHAP values appear to be more spread out around the zero point in model 2 which might suggest that serial 7’s scores are more strongly correlated with physical mobility than CDT scores in DTWT test.

## 4 Discussion

This study investigated the gait and turn features of NT older adults and patients with cognitive impairment using a novel test design and dual Kinect sensor set up. To our knowledge this is the first study to analyze walk and turn dynamics in patients with cognitive impairment. Our results revealed that turn features were notably different among groups even after considering the effects of covariates and they also appeared to be more strongly connected to cognitive function than gait-related measures.

### 4.1 Significance of gait and turn metrics

DTWT test results were in line with previous studies showing the effect of an additional cognitive task on gait performance (Ramirez and Gutierrez, 2021). Gait speed and stride time were the most significant factors in distinguishing the AD, aMCI, and NT groups, with a notable effect size. This is consistent with the findings of review studies which marked the dual-task walking speed as a deterministic gait characteristic influencing individuals with cognitive impairment (Koppelmans et al., 2022; Cepukaityte et al., 2024). Research indicates that gait speed is associated with reduced hippocampal volume, thinner temporal gyrus, and alterations in PFC activation (Callisaya et al., 2013; Koppelmans et al., 2022). Among patients with AD, our study revealed a greater STV which has an association with higher cortical brain control, suggesting that cognitive decline may lead to increased STV (Pieruccini-Faria et al., 2021).

Regarding turn performance, in healthy older adults limited range of motion due to balance issues were reported which could serve as a compensatory mechanism to enhance stability (Baird and Van Emmerik, 2009; Khobkhun et al., 2022b). However, our findings revealed a more pronounced issues in patients with cognitive decline in the presence of a cognitive load. Turning requires reorienting the body’s CoM, coordinating segmental rotations and adjusting step placement and demands more balance and coordination. Turning also, engages a wider range of brain regions compared to straight walking, semi-automatic once initiated and primarily involves subcortical structures, especially because of the need to rapidly coordinate movements while processing spatial information (Miri et al., 2024). Executive functions, such as planning, attention, and sequencing, are crucial during turning as individuals must plan and execute the turn without losing balance (Mirelman et al., 2017; Stuart et al., 2018). In addition to balance and coordination issues, navigation relies on distinct neural pathways, particularly the medial temporal lobes, including the hippocampus and entorhinal cortex, are essential for processing visuospatial information which is vital when navigating a turn (Shadmehr and Krakauer, 2008; Takakusaki, 2017). Moreover, turning involves in proprioception and the integration of sensory information to update body orientation in space and determining the trajectory, especially when avoiding obstacles which would necessitates the involvement of parietal lobe (Cohen and Andersen, 2002; Koppelmans et al., 2022). Deficits in these regions, leads to impairments in both spatial navigation and turning performance. For instance, longer turning time in DTWT test observed in patients with cognitive impairment is align with the recent study’s discovery attributing this to the reduced volume of the hippocampus (Wang et al., 2024). Moreover, patients with aMCI and AD had shorter step length and lower body segment speeds compared to the NT group.

Based on the observations we hypothesize that the results may be attributed to the dysfunction in the allocentric-to-egocentric transitions during spatial processing. Allocentric navigation involves understanding one’s position relative to the environment, represented as a map, which is crucial for navigation and path integration. The medial temporal lobe, particularly the entorhinal cortex and hippocampus, are recognized as the primary brain regions involved in allocentric processes (Cepukaityte et al., 2024; Chrastil, 2025). Whereas egocentric navigation relies on medial parietal regions, namely the retrosplenial cortex, to maintain awareness of one’s position in relation to immediate surroundings, often necessitating rapid adjustments and motor responses (Epstein et al., 2017; Cepukaityte et al., 2024). This process depends on the ability to determine the spatial relationships between different body parts and external objects, which is directly reinforced by proprioceptive feedback. The brain regions mentioned earlier, vital for spatial navigation, are affected in the early stages of AD due to the deposition of amyloid and tau pathology found in the initial Braak pathological staging (Braak and Braak, 1991; Levine et al., 2020; Cepukaityte et al., 2024). While first perceived as a purely motoric action, making a turn requires the perception and integration of information on the orientation of body segments in relation to each other and the environment in order to navigate on a predetermined course. Given that certain early aspects of AD pathology are also involved in the navigation circuitry, the deficits in turning can be elucidated. In addition, white matter atrophy could disrupt the connectivity between cognitive and motor regions and basal ganglia dysfunction could complicate motor control due to its role in regulating voluntary motor movements and procedural learning (Koppelmans et al., 2022; Xiong et al., 2022).

Along with the preexisting problems and additional spatial processing, the presence of a cognitive load will lead to the adoption of more cautious strategies while making a turn. For instance, a decreased peak segmental angle indicates that individuals with AD have an en-bloc turn similar to those with Parkinson’s disease. Prior research has shown comparable findings in older adults during a turn as a tactic to simplify the turning process and compensate the decrease in stability and balance (Baird and Van Emmerik, 2009; Khobkhun et al., 2021; Khobkhun et al., 2022a). Meanwhile, research that employed the dual-task test in a straight line revealed those with cognitive impairment had altered brain activation in the PFC and increased functional connectivity, leading to worse gait performance (Weng et al., 2023). Thus, the en-bloc pattern of reorientation in persons with AD upon the DTWT test is more pronounced due to their pre-existing deficits in brain regions associated with spatial navigation and decreased activity of the PFC resulting from the cognitive load. The further approach to preserve balance during a turn observed in individuals with AD is the decrease of sway-AP. In essence, the AD group had to relocate their CoM to the terminal point of the pathway in order to effectively execute a turn, which also led to a decrease in turn speed.

These biomechanical and cognitive changes in turning performance have important real-world implications, particularly regarding fall risk. Indeed, deficits in turn performance, especially during cognitively demanding tasks, are often associated with an increased risk of falls in older adults (Almajid et al., 2020). Given that turn performance is a critical component of mobility, the observed difficulties in executing turns among cognitively impaired individuals could be an early indicator of fall risk. While our study did not directly assess fall occurrences, the changes in step length, turning time, and sway are consistent with those reported in fall-prone populations (Cheng et al., 2014; Gulley et al., 2020).

### 4.2 Association between cognitive performance and walk and turn features

Previous research examining the relation between cognitive performance and gait characteristics largely employed the trail making test to assess executive function and the digit span forward test to evaluate working memory (Doi et al., 2017; Weng et al., 2023; Poosri et al., 2024). However, to investigate the interplay between cognitive capacity and motor function, the CDT and serial 7s tests were selected test because, the act of walking and turning while simultaneous counting backwards is a complex procedure that demands the participation of several cognitive domains, including planning, visuospatial ability, and attention (Mirelman et al., 2014), which are assessed by the CDT. Typically employed as a cognitive task in the dual-task test, the serial 7s test assesses not only working memory but also attention and the capacity to update and recall information during calculations (Schneider, 1983). This cognitive process is similar to the needed mental updating during turning.

As for gait features, gait speed and stride time are found to be associated with working memory. This is consistent with prior research suggesting that working memory and gait speed are predominantly mediated by the same regions of the brain and the simultaneous cognitive processing in these regions may lead to a limitation in brain resources that impacts the walking ability of individuals with MCI (Montero-Odasso et al., 2009a). Meanwhile, stride length is significantly linked to the CDT test suggesting that this characteristic requires planning and visuospatial abilities (MacAulay et al., 2015), particularly in our scenario where there is a limited path and the length of the steps must be planned. We propose a hypothesis that the step length during a turn is connected with the CDT test in a similar way.

Our findings suggest a notable association between body segmental angle and CDT test. Executing a turn is a sequential procedure that begins with turning the head first, followed by the chest and hips (Khobkhun et al., 2022b). This could be similar to sequential steps while performing a CDT test explaining the positive relationship with segmental angle and CDT scores which indicates decline in performing a sequential activity in individuals with cognitive impairment. Supporting previous research, in our results peak head speed, peak hip speed, and turning time exhibit a correlation with working memory, same as gait speed, which emphasized on the correlations between processing speed and prefrontal lobe function (Mancini et al., 2016; Doi et al., 2017). Turning and counting backwards would restrict the cognitive resources available in PFC, making counting and keeping balance a struggle resulting in reduced speed.

Overall, the results have illuminated the complex interplay between cognitive functions and motor tasks, particularly turning, which requires higher-order cortical engagement. Utilizing the CDT and serial 7s tests, our analysis underscores the pivotal roles of planning, visuospatial abilities, and working memory in managing turning tasks with additional cognitive loads. Our findings reveal that turning, as opposed to straight walking, demands more extensive use of PFC resources, as turn-related parameters—such as segmental angles, peak speeds, and step length—demonstrated stronger correlations with cognitive test scores than did traditional gait measures. This suggests a more substantial cognitive demand for turning, which could serve as a sensitive indicator of early cognitive decline far more than linear gait metrics.

### 4.3 Strengths and limitations of the study

This study had several strength and limitation. The performed test was non-invasive because no sensor or device was attached to the participants. Also, common devices for gait analysis are often expensive and unavailable for some clinical centers thus, we used affordable and clinically valid Kinect sensors (Springer and Seligmann, 2016). However, single Kinects could be inaccurate at capturing lower limb joints and curved movements, hence experts recommend the dual Kinect system, as employed in this study (Kotsifaki et al., 2018). Additionally, the simple cognitive task effectively evaluated patients with lower levels of education.

Gait and turn assessment require musculoskeletal health and no serious medical issues in older adults, making it unavailable to all. Our investigation did not detect significance in gait features like symmetry, which have been found in earlier research, potentially due to the limited field of view of the Kinect cameras not providing enough walking distance to identify the difference among groups. Also, due to resource constraints, this study enrolled a moderate sample size of 75 participants, which was calculated beforehand to ensure sufficient power, however, it fell short of the larger sizes. It is worth noting that age and education levels differed between groups. To address these differences and their effects on independent variables, we applied the ANCOVA test with age and years of education as covariates, ensuring that our main comparisons were not confounded by these demographic factors which is a method applied by similar studies (Montero-Odasso et al., 2017; Pieruccini-Faria et al., 2021; Bovonsunthonchai et al., 2022).

To validate and expand these findings, future research should aim for incorporating larger sample sizes and extended pathways. Longitudinal could enhance the prognostic efficiency of turn dynamics for detecting cognitive impairment. Moreover, exploring whether deficits in turning dynamics, as seen in patients with aMCI and AD, are predictive of fall incidents, could provide a potential screening tool for identifying those at risk.

## 5 Conclusion

This study explored the diagnostic potential of the walk-and-turn test with emphasize on turn variables. Our results suggest that, in addition to routine gait parameters, turn dynamics could serve as newer, more sensitive markers for the early detection of cognitive decline in older adults.

## Data Availability

The raw data supporting the conclusions of this article will be made available by the authors, without undue reservation.
