# Supplementary material for: Talking during walking: the diagnostic potential of turn dynamics in Alzheimer’s disease, mild cognitive impairment and cognitive aging
Source: Front Aging Neurosci. 2025 Feb 19;17:1533573. doi: 10.3389/fnagi.2025.1533573 (PMC11880252; doi:10.3389/fnagi.2025.1533573)
Supplement: Supplementary file 1 [file Table_1.docx]

Supplementary Material

**Data recording and acquisition procedures with double Kinect setup**

Calibration procedure:

The calibration process illustrated in eFigure 1 included waving a standard-sized board in the field of view of both sensors for 20 seconds and perform the calibration process on the iPi motion studio software to locate the sensors’ positions in space.


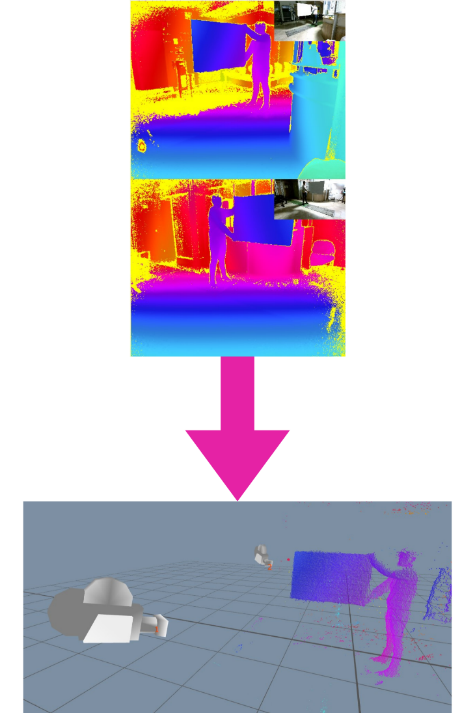


eFigure 1 Calibration procedure

Synchronization:

To guarantee synchronized data capture from the cameras, they were linked to laptops on a shared wireless network. One laptop was designated as the master, which initiated and terminated both cameras' recording sessions.

Skeleton data extraction:

Post-capture, the recorded data from the two Kinect sensors were merged using the iPi Recorder software. Subsequently, the merged files were then imported into iPi Studio for the motion tracking process. The software tracked human body movements and generated the skeleton data using the Bio-mech add-on of iPi Studio. To ensure the smoothness of the tracked movements, jitter removal and refinement processes were applied. As demonstrated in eFigure 2.


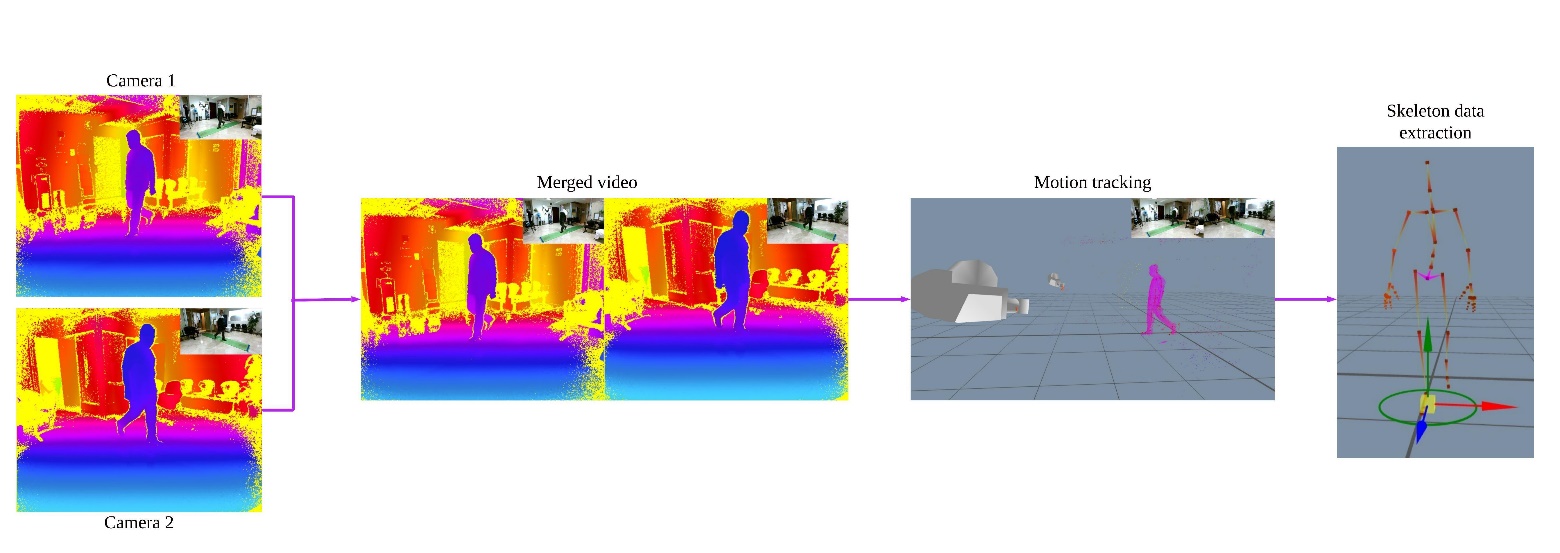


eFigure 2 Motion tracking and skeleton data extration

**walk phase variables definitions**

eTable 1 Walk phase variables

| **Variable** | **Definition** |
| --- | --- |
| stride length | distance between two consecutive heel points of footfalls of the same foot |
| stride time | duration taken for one stride length |
| gait speed | distance covered over time |
| double support time (DST) | duration of when both limbs are in contact with the ground |
| cadence | number of steps by time |
| stride time variability (STV) | [(standard deviation of stride time/mean of stride time) $\times$100] |
| swing phase | average time in which one foot is in contact with the floor |
| stance phase | average time with two feet touching the ground |

**
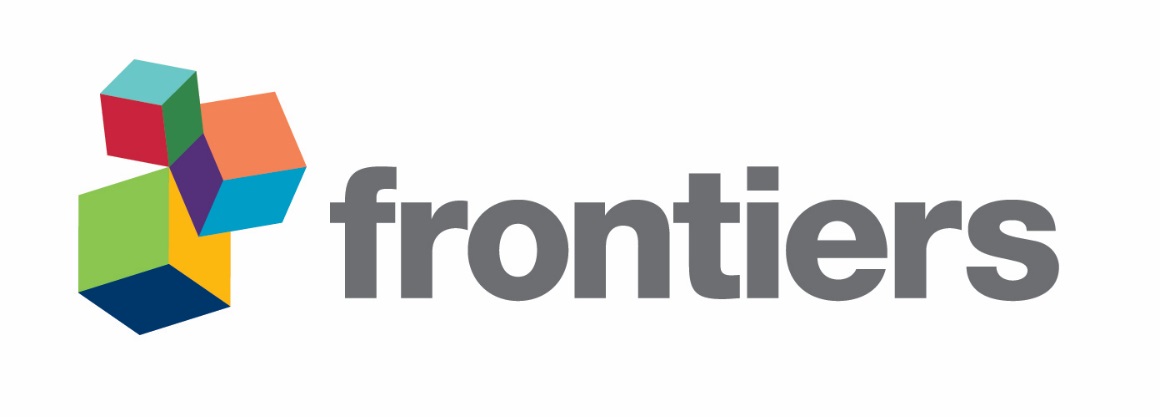
**
